# Exploring response signals and targets in aggressive unresectable hepatocellular carcinoma: an analysis of targeted therapy phase 1 trials

**DOI:** 10.18632/oncotarget.4601

**Published:** 2015-06-23

**Authors:** Ishwaria M. Subbiah, Gerald S. Falchook, Ahmed O. Kaseb, Kenneth R. Hess, Apostolia M. Tsimberidou, Siqing Fu, Vivek Subbiah, David S. Hong, Aung Naing, A. Piha-Paul Sarina, Owais Akmal, Filip Janku, Razelle Kurzrock

**Affiliations:** ^1^ Division of Cancer Medicine, University of Texas MD Anderson Cancer Center, Houston, TX, USA; ^2^ Department of Investigational Cancer Therapeutics, University of Texas MD Anderson Cancer Center, Houston, TX, USA; ^3^ Department of Gastrointestinal Medical Oncology, University of Texas MD Anderson Cancer Center, Houston, TX, USA; ^4^ Department of Biostatistics, University of Texas MD Anderson Cancer Center, Houston, TX, USA; ^5^ Moores Cancer Center, University of California San Diego, San Diego, CA, USA

**Keywords:** targeted agents, novel therapeutics, management, systemic therapy, clinical trials

## Abstract

**PURPOSE:**

Patients with advanced hepatocellular carcinoma (HCC) have limited effective therapeutic options. Given the rapid advanced in drug development and emergence of novel agents, we analyzed the characteristics and outcomes of HCC patients treated on early phase trials with an emphasis on targeted therapies.

**METHODS:**

We reviewed the records of consecutive HCC patients evaluated in the Phase I Clinical Trials Program at MD Anderson from March 2004.

**RESULTS:**

Thirty-nine patients were not treated due to poor performance status (*n* = 22, 56%) and decision to pursue alternate therapies (*n* = 10, 27%). Of 61 treated patients (median age, 60 years; median prior therapies, 3), eight patients (13%) attained stable disease lasting ≥6 months; four (7%) had a partial response, mainly with anti-angiogenic or multikinase inhibitors. Median Phase I progression-free survival (PFS) was 2.6 months versus 4.4 months (*p* 0.019) and 4.1 months (*p* 0.27) for their first-, and second-line FDA-approved therapy. Molecular analysis showed frequent *PTEN* loss (10/19 patients, 53%) and *P53* mutation (4/4 patients tested). On multivariate analysis, independent factors predicting shorter survival were white ethnicity/race (p 0.031), cirrhosis (*p* 0.016), and serum sodium (*p* 0.0013).

**CONCLUSIONS:**

In our heavily-pretreated HCC patients, the phase I PFS was comparable to that of 2^nd^-line therapy, highlighting a potential role for clinical trials after progression on first-line therapy. The response rate (SD>6 months/PR) of 20% was observed with early signals of activity in regimens combining inhibitors of angiogenesis, multiple kinases and mTOR with preliminary molecular analysis revealing prevalence of *PTEN* loss.

## INTRODUCTION

Advanced hepatocellular carcinoma (HCC) remains a therapeutic challenge with limited effective treatment options. Patients with limited localized disease are considered for surgical resection or liver transplantation, both of which remain the only potentially curative options. [1-3] However in patients with recurrent or unresectable disease, the mainstay of therapy remains monotherapy with sorafenib, an oral multikinase inhibitor of vascular endothelial growth factor (VEGF), platelet derived growth factor receptor (PDGFR) and RAF.[4, 5] The pivotal multicenter, phase 3, double-blind, placebo-controlled trial in 602 patients with advanced HCC demonstrates an improvement in the median overall survival (10.7 months) in the sorafenib group versus the placebo group (7.9 months).[6] Multimodal therapeutic approaches using arterial embolization or chemoembolization also demonstrate a survival benefit over supportive care alone in patients with unresectable disease.[7, 8] Recent advancements incorporated the use of radioembolization with yttrium-90 into the treatment algorithm for intermediate to advanced HCC with Child-Pugh class A or B.[9-11] With the expanding development of agents targeting angiogenesis and aberrant cell signaling, we explored the characteristics and outcomes of patients with advanced HCC who received treatment on a phase I clinical trial with an emphasis on targeted therapies.

## RESULTS

### Patients characteristics

Of the 100 patients with HCC referred to the Phase I Clinical Trials program, 39 patients were not enrolled in a phase I clinical trial due to poor performance status (ECOG >3, *N* = 22, 56%), decision to pursue alternate therapies including treatments closer to home (*N* = 10, 26%), no evidence of disease post-resection (*N* = 3), prohibitive lab abnormalities (*N* = 2) and insurance denial (*N* = 2).

Overall, 61 patients who participated in a phase I trial are included henceforth in this analysis. Pretreatment characteristics at presentation to the Phase I clinic are summarized in Table 1. The median age at diagnosis was 60 years (range, 11-84 years). There were 12 women and 49 men of whom 30 (49%) were White, 11 (18%) Asian, 10 (16%) African American, and 10 (16%) of Hispanic origin. Eleven patients (18%) had an ECOG PS of 0, 49 patients (80%) had a PS of 1 and 1 (2%) patient a 2. The median number of metastatic sites was 2 (range 0-5). The most common sites of metastases at time of Phase I referral were liver (*N* = 49, 80% of patients), lymph nodes (*N* = 31, 51%), lung (*N* = 25, 41%), peritoneum (*N* = 14, 23%), bone (*N* = 13, 21%), adrenal (*N* = 8, 13%), and other (ovary, pancreas, spleen; *N* = 3, 5%). Regarding the extent of liver disease, 41 patients (37%) were classified as modified Child-Pugh class A while the remaining 20 (33%) had Class B disease.

### Therapy before enrollment on phase I trials

Overall, of the 61 patients enrolled on a phase I trial, four patients had received no prior therapy because of the unavailability of reasonable, conventional therapy for the extent of their disease or patient desire to pursue clinical trial. The remaining 57 patients had a median of 3 prior systemic therapies before referral to the phase I clinic (range, 1 – 8). Twenty six patients (43%) also underwent a prior chemoembolization, 17 (28%) a surgical resection, 12 (20%) yttrium-90 radioembolization, 12 (20%) radiation, 8 (12%) a radiofrequency ablation, and 3 (5%) an orthotopic liver transplantation.

For their first-line treatment in the advanced/metastatic setting, 41 of 61 patients received a sorafenib-based regimen, of whom 39 received single-agent sorafenib while 2 received sorafenib in combination with erlotinib. Three patients received erlotinib plus bevacizumab; 5 received a capecitabine-based regimen, 4 received a gemcitabine-based regimen, 3 received platinum combination therapy, and 1 received experimental therapy on phase II trial with a novel camptothecin analog. 33 patients did not receive a second line treatment with FDA-approved agents and instead proceeded with a phase I clinical trial.

### Treatment

Overall, patients were initially treated on 1 of 31 different phase I clinical trials; of these trials, 19 patients received therapy on 11 “first-in-human” trials with novel targeted inhibitors against VEGFR, kit, cMET, EGFR, HER2, CHK1, RAS, organic arsenic or an oleander extract derivative.

Of 61 patients, 39 (64%) were treated on a trial with combination therapy of two or more agents while 22 patients (36%) received treatment with a single agent. Ten (16%) patients received liver-directed treatment with direct infusion of a cytotoxic agent into the hepatic artery, of which 2 as a single agent and 8 in combination most commonly with bevacizumab. 37 (61%) received an oral or intravenous angiogenesis inhibitor, 6 (10%) of whom received the anti-angiogenic agent in combination with liver-directed chemotherapy. Forty-nine patients (80%) received targeted agent(s) alone, nine (15%) received targeted agent(s) in combination with cytotoxic chemotherapy, and three (5%) received cytotoxic chemotherapy alone. The median number of cycles received was two (range 1 – 28). Eighteen patients went on to receive therapy under a second phase I trial; of these patients, 11 received treatment on 3 phase I trials, 2 were treated on a 4^th^ trial.

**Table 1:** Patient characteristics

		N	%
**Gender**			
	Male	49	80%
	Female	12	20%
**Age at time of diagnosis**			
	Median (range)	60.2	(11.3-83.5)
**Race/Ethnicity**			
	White	30	49%
	Asian	11	18%
	Hispanic	10	16%
	African American	10	16%
**ECOG Performance Status**			
	0	11	18%
	1	49	80%
	2	1	2%
**Underlying liver pathology**			
	Hepatitis C	20	33%
	Alcohol abuse	16	26%
	Hepatitis B	20	33%
	Steatohepatitis	11	18%
	Autoimmune hepatitis	3	5%
	Hemochromatosis	1	2%
	None	7	11%
**Comorbidities**			
	Diabetes	16	26%
	Hyperlipidemia	9	15%
	Coronary artery disease	8	13%
**Imaging characteristics**			
	Presence of cirrhosis	30	49%
	Presence of ascites	21	34%
	Portal hypertension	17	28%
	Portal vein thrombosis	29	48
	Lobar distribution		
	Unilobar	25	41%
	Bilobar	36	59%
	Distribution		
	Solitary	6	10%
	Multifocal	55	90%
**# of metastatic sites, median (range)**		2	(0-5)
**Metastatic sites**			
	Liver	49	
	Lymph nodes	31	
	Lung	25	
	Peritoneum	14	
	Bone	13	
	Adrenal	8	
	Other	3	

### Response

**Figure 1:**
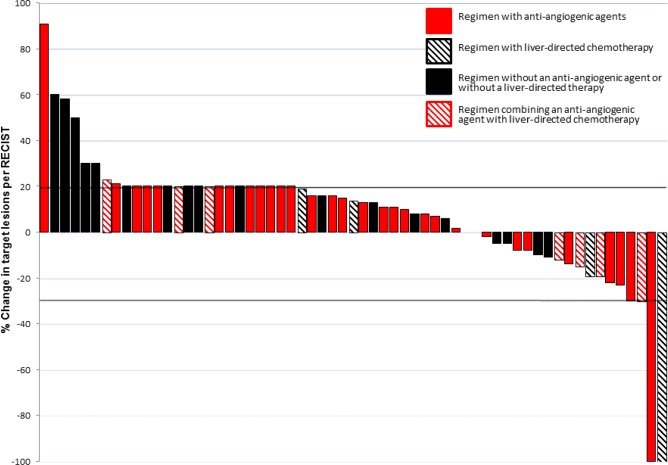
Waterfall plot showing the best responses to phase I therapy per RECIST

Fourteen patients were not restaged prior to end of cycle 2 due to clinical deterioration and early disease progression and are identified on the waterfall plot as having a 20% increase in tumor size (Figure 1). Of the 61 patients treated on studies, 4 (7%) had a partial response (PR), 23 had stable disease (38%) including 8 (13%) who had SD > 6 months, with a SD > 6 months/PR rate of 20% (12 of 61 patients); 10 of these 12 patients received an oral or IV inhibitor of angiogenesis. The characteristics and specific treatment regimens of these responders are detailed in Table 2. Thirty-five patients (57%) had progressive disease (PD). The highest response rates (SD > 6 months/PR) were observed in patients treated with protocols that included agents targeting angiogenesis and mTOR. Of 10 patients who were treated on an HAI-based protocol, 3 (30%) has a significant clinical response including two with prolonged SD > 6 months and 1 PR.

**Table 2:** Characteristics of responses (PR + SD > 6 months)

Pt #	Age/Sex	Phase I Regimen	RECIST response	% change in target lesions per RECIST	Phase I PFS (m)
**1**	54/M	Bevacizumab + sorafenib	SD	−8	9.5
**2**	63/F	HAI Oxaliplatin + IV 5FU, LV + bevacizumab	SD	−15	11.8
**3**	74/F	Bevacizumab + sorafenib	SD	−14	10.4
**4**	56/F	Regorafenib	PR (CR of target lesions; stable bone mets)	−100	27.0+
**5**	56/M	Regorafenib	SD	10	7.8
**6**	73/M	Novel inhibitor of HDAC, EGFR1, and Her2	SD	6	11.1
**7**	57/M	HAI paclitaxel	PR (CR of target lesions; stable non-target previously embolized liver lesions)	−100	41.3
**8**	49/M	HAI Oxaliplatin + IV 5FU, LV + bevacizumab	PR	−30	6.8
**9**	58/M	Regorafenib	SD	8	7.5
**10**	23/M	Bevacizumab + sorafenib	SD	−23	7.4
**11**	46/F	Pazopanib + everolimus	PR	−30	8.3
**12**	68/M	Bevacizumab + bortezomib	SD	2	6.2

Abbreviations: EGFR epidermal growth factor receptor, FU fluorouracil, HAI hepatic arterial infusion, LV leucovorin.

One dramatic response was observed in a 56-year old Asian woman with metastatic HCC in setting of hepatitis C, Child-Pugh class A. Her prior therapies included sorafenib monotherapy and then a combination of bevacizumab plus erlotinib. Upon progression, she then enrolled on phase I therapy with regorafenib, then a novel oral small molecular inhibitor of VEGFR2 and 3, and Ret, Kit, PDGFR and Raf kinases. She demonstrated a complete response of target lesions with stable non-measurable bone lesions, hence deemed a partial response per RECIST, and has maintained this remarkable response for 27 months ongoing. A second response was observed in a 57-year old man who received HAI paclitaxel; he demonstrated a complete response of target liver lesions with stable post-embolization non-target hepatic masses, thereby attaining a partial response.

### Survival and toxicities

**Figure 2:**
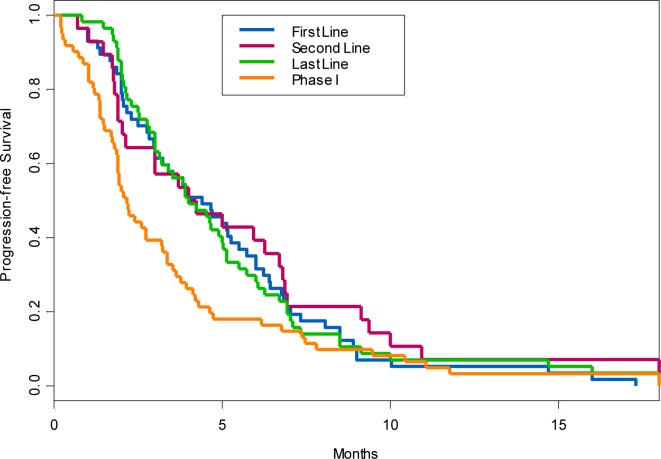
Progression-free survival of patients treated on phase I trials when compared to their first-line, second-line and last systemic antitumor therapy given in advanced setting prior to phase I referral

The median time from diagnosis of advanced/metastatic disease to date of primary evaluate in the phase I clinic was 9.6 months for the 61 patients who enrolled on a phase I trials. The median time from the primary phase I consultation to beginning therapy on a phase I clinical trial was 19 days. The median overall survival from day one on a phase I trial was 7 months (95% CI 6, 13). Median PFS for 61 treated patients was 2.6 months (95% CI 1.9, 3.4) on phase I clinical trials. Among these 61 patients, the median PFS on their first-line and second-line prior therapies with FDA approved agents given in the advanced setting was 4.4 months and 4.1 months, respectively. In comparison, the PFS on the first line FDA-approved therapy given in the advanced setting prior to Phase I referral was improved compared to the PFS on phase I therapy (*p* 0.019). However, the second-line therapy with FDA-approved drugs was comparable to the PFS on a phase I trial (*p* 0.27). The PFS on first-, second-line and Phase I therapy are shown in Figure 2.

Among the 61 treated patients, 42 (69%) had died at the time of analysis. The 90-day mortality was 38% with 38 patients alive at 3 months after beginning phase I therapy; the 6-month mortality was 61% with 24 patients alive at 6 months after beginning therapy on phase I trials. Importantly, there was no treatment-related mortality. One patient treated on a combination regimen that included sorafenib experienced grade 3 hand foot syndrome that was not responsive to a dose reduction. This patient ultimately showed disease progression on restaging imaging. A second patient developed a mild headache, dizziness and left-sided visual field blurriness five days after beginning a sunitinib-based combination therapy and was found to have a small right parieto-occipital intracranial hemorrhage, possibly related to therapy. There were no other high-grade toxicities reported.

### Prognostic factors for survival

We conducted univariate and multivariate analysis to evaluate the effects on survival of variables including age, sex, race/ethnicity, ECOG performance status, risk factors for liver disease (alcohol abuse, hepatitis C, hepatitis B), comorbidities (coronary artery disease, type II diabetes), extent of liver disease (presence of cirrhosis, portal hypertension, ascites, portal vein thrombosis), alpha-fetoprotein; history of thromboembolism; number of prior therapies; presence of liver metastases; number of metastatic sites; hemoglobin level; platelet count; and albumin, lactate dehydrogenase (LDH), alkaline phosphatase, bilirubin, alanine aminotransferase, aspartate aminotransferase, sodium, and creatinine levels (Table 3). Predictors of shorter Phase I PFS in univariate analysis were the presence of cirrhosis (*p* 0.016), portal hypertension (*p* 0.011), ascites (*p* 0.012), abnormal sodium (*p* 0.028) and hypoalbuminemia (0.0013); these five factors also predicted for shorter overall survival (*p* 0.012, 0.038, 0.013, 0.031, and <0.0001 respectively). The multivariate analysis showed cirrhosis (*p* 0.038), portal hypertension (*p* < 0.017), and serum sodium (*p* 0.0012) as predictors of shorter PFS on phase I therapy while cirrhosis, white race and abnormal serum sodium predicted for a shorter OS (Table 4).

**Table 3:** Summary of univariate analysis

	Association with PFS		Association with OS	
	Hazard ratio (95% CI)	*P*-value	Hazard ratio (95% CI)	*P*-value
Gender	0.7 (0.4, 1.4)	0.31	0.8 (0.3, 1.7)	0.51
Age at diagnosis	1.3 (0.8, 2.1)	0.35	1.6 (0.9, 2.9)	0.14
ECOG PS 0 vs. 1-2	0.8 (0.4, 1.5)	0.44	1.0 (0.5, 2.1)	0.95
Ethnicity/Race (White vs. non-white)	1.2 (0.7, 2.0)	0.47	2.0 (1.0, 3.9)	**0.039**
**Risk factors for chronic liver disease**				
Alcohol abuse	0.9 (0.5, 1.6)	0.66	0.6 (0.3, 1.2)	0.12
Hepatitis C	1.0 (0.6, 1.8)	0.99	0.7 (0.4, 1.5)	0.38
Hepatitis B	1.2 (0.7, 2.2)	0.47	1.5 (0.8, 2.9)	0.2
**Other comorbidities**				
Coronary artery disease	1.6 (0.7, 3.4)	0.27	1.7 (0.7, 4.4)	0.3
Type II diabetes	0.7 (0.4, 1.3)	0.24	0.8 (0.4, 1.7)	0.6
**Extent of chronic liver disease**				
Cirrhosis	1.9 (1.1, 3.2)	**0.016**	2.2 (1.2, 4.0)	**0.012**
Portal hypertension	2.2 (1.2, 4.0)	**0.011**	2.2 (1.1, 4.4)	**0.038**
Portal vein thrombosis	1.1 (0.7, 1.8)	0.69	1.2 (0.6, 2.2)	0.57
Ascites	2.1 (1.2, 3.6)	**0.012**	2.4 (1.2, 4.5)	**0.013**
**Extent of HCC**				
# of metastatic sites	1.2 (0.7, 2.0)	0.47	1.5 (0.8, 2.8)	0.19
# of prior therapies	0.8 (0.5, 1.4)	0.51	1.0 (0.5, 1.8)	0.96
**Baseline laboratory values**				
Anemia (Hg <10.5g/dL)	0.8 (0.4, 1.6)	0.6	1.3 (0.6, 2.8)	0.57
Elevated LDH (>618IU/L)	1.4 (0.8, 2.4)	0.23	1.9 (1.0, 3.5)	0.051
Thrombocytopenia (<158K/UL)	0.9 (0.6, 1.6)	0.8	1.1 (0.6, 2.0)	0.8
Elevated alpha-fetoprotein (>120)	1.2 (0.7, 2.0)	0.5	1.5 (0.8, 2.8)	0.22
Elevated total bilirubin (>1mg/dL)	1.5 (0.8, 2.7)	0.22	2.3 (1.2, 4.6)	**0.025**
Elevated INR (>1.1)	1.5 (0.9, 2.6)	0.1	1.4 (0.8, 2.7)	0.23
Elevated ALT (>56IU/L)	1.0 (0.6, 1.8)	0.97	0.8 (0.4, 1.5)	0.45
Elevated AST (>46IU/L)	1.5 (0.7, 3.0)	0.26	2.0 (0.7, 5.5)	0.16
Alkaline phosphatase >126IU/L	1.5 (0.8, 2.9)	0.24	1.6 (0.7, 3.5)	0.24
Abnormal serum sodium	2.8 (1.2, 6.4)	**0.028**	3.0 (1.2, 7.4)	**0.031**
Serum albumin <3.5g/dL	2.8 (1.5, 5.1)	**0.0013**	5.2 (2.4,11.2)	**<0.0001**
Serum creatinine >1.3mg/dL	NR	n/a	NR	n/a

Abbreviations: ALT Alanine aminotransferase; AST Aspartate aminotransferase; ECOG Eastern Cooperative Oncology Group; LDH Lactate dehydrogenase; NR not reached; PS Performance status.

**Table 4:** Summary of multivariate analysis

	Hazard ratio	Lower limit	Upper limit	*P* value
**Analytic variable associated with shorter PFS**				
Cirrhosis	1.8	1	3.1	0.038
Portal hypertension	2.2	1.2	4.2	0.017
Abnormal serum sodium	4.3	1.8	10.3	0.0012
**Analytic variable associated with shorter OS**				
White race	2.2	1.1	4.6	0.031
Cirrhosis	2.3	1.2	4.4	0.016
Abnormal serum sodium	4.9	1.9	13	0.0013

Abbreviations: OS overall survival; PFS progression-free survival.

### Molecular analysis

Testing for mutations in *KRAS, NRAS, BRAF, CKIT, EGFR, PIK3CA, TP53, MET, GNAQ, AKT1, ALK* rearrangement as well as immunohistochemistry for *PTEN* loss, ER, and *ALK1* and *HER-2/neu* and *MET* amplification via FISH was completed on patients with adequate available tissue in the MD Anderson CLIA-certified laboratories. On immunohistochemistry, 10 of 19 tested patients demonstrated an abnormality in *PTEN* expression with 7 tumors showing frank *PTEN* loss and 3 samples showing very faint *PTEN* expression (<10% of stained cells). PCR-based DNA sequencing analysis was performed in exons 4 to 9 of the *TP53* gene. The lower limit of detection is approximately one cell bearing the mutation per five cells (20%). All 4 tested patients demonstrated a *TP53* aberration, one patient with a 12 base pair deletion in exon 4 and 3 patients with a missense mutation in codon 177 in exon 5 (P177L), codon 158 in exon 5 (R158H), and codon 272 in exon 8 (V272M). *KIT, PIK3CA,* and *MET* mutations were found in individual patients. The remainder of the mutational analyses was negative including *KRAS* (all 19 tested), *PIK3CA* (17 of 18 tested), *BRAF* (all 17 tested), and *EGFR* (all 13 tested).

## DISCUSSION

Unresectable hepatocellular carcinoma in the setting of Child-Pugh class A or B liver disease remains a clinical challenge particularly after relapse on sorafenib given in the metastatic setting. Alternate recommended options include locoregional therapy with transarterial chemoembolization, conformal or stereotactic radiation, or clinical trial.[13] Multimodal approaches include liver-directed therapies such as embolization with the radionuclide yttrium-90.[14] However, for the patient with extrahepatic disease, the need for novel targeted systemic therapies and combination regimen is self-evident.

In our analysis, patterns of early signals suggesting clinical activity emerged in regimens that target angiogenesis particularly in combination with multikinase inhibition using sorafenib. Of particular interest is the observation of clinical response using a combination of bevacizumab and sorafenib in our HCC patients who have all previously received single-agent sorafenib. These patients had demonstrated disease regression with sorafenib monotherapy given as frontline agent in the metastatic setting but subsequent development of secondary resistance with disease progression. This concept of successful retreatment after initial response and subsequent progression has been explored in small case series and provides an intriguing challenge to the prevailing convention that a resistant tumor is unlikely to respond to a rechallenge with a similar regimen.[15, 16] Additional response signals were observed in regimens utilizing intravenous angiogenesis inhibition in combination with liver-directed infusion of cytotoxic chemotherapy. Indeed ten of the twelve prolonged responders received an angiogenesis inhibitor, of which two were in combination with hepatic arterial infusion therapy.

For the clinician faced with the relapsed advanced HCC patient, the timing of referral for a clinical trial remains essential point of decision. In our analysis, frontline therapy with sorafenib given in the advanced setting prior to phase I referral had an improved PFS in comparison to the PFS in phase I clinical trial. However the PFS on the second line therapy with FDA-approved agents given in the advanced setting prior to phase I referral was comparable to the PFS on phase I trial. Furthermore, in our analysis, of the 100 patients referred for phase I trials, 39% were ineligible mainly due to decline in performance status (ECOG PS of 3 or greater) and prohibitively abnormal laboratory values, underscoring the importance of early participation in clinical trials after progression on frontline therapy in the metastatic setting.

Overall, the pattern of molecular aberrations seen in HCC is slowly emerging. In our analysis, the most common molecular aberration was observed in the expression of the tumor suppressor gene *PTEN*. We observed complete loss or very faint *PTEN* expression in 10 of 19 (53%) of tested tumor samples, highlighting the therapeutic target potential of the PI3K/AKT/mTOR pathway. Indeed activation of the mTOR signaling has been demonstrated in HCC where immunohistochemical analysis shows significantly elevated expression of p-mTOR in the sinusoidal endothelial cells of HCC tissue samples when comparison with non-cancerous tissue such as normal liver, cirrhotic nodules, or hepatic adenomas.[17, 18] Indeed, preclinical murine models of HCC have demonstrated a greater degree of carcinoma growth inhibition with the combination of bevacizumab plus rapamycin treatment rather than monotherapy with either agent.[19] Furthermore, mTOR inhibition with rapamycin has been explored in the context of decreased post-transplant tumor recurrence in patients receiving sirolimus for immunosuppression.[20] Another meta-analysis investigated 2950 patients with resectable HCC who underwent a liver transplantation; among these patients, those who were placed on a sirolimus-based regimen for immunosuppression had a decreased risk for disease recurrence and an improvement in their overall survival, highlighting the significant dual role of mTOR inhibitors in this selected population.[21]

Another emerging molecular aberration is mutations in the *TP53* gene encoding the tumor suppressor protein P53. Polymorphisms particularly in codon 72 of *TP53* have been associated with increased risk for multiple solid malignancies including high-grade serous ovarian and pancreatic adenocarcinoma.[22, 23]

In one series, *TP53* mutations have been associated with recurrence of HCC; indeed, 16 of 33 cases (49%) of recurrent HCC were observed to harbor the *P53* mutation; additionally patients whose tumor had the *P53* mutation had a faster time to recurrence after surgical resection, further supporting this mutation’s role in carcinogenesis.[24]

Our analysis demonstrates several limitations. First, a selection bias exists within our patient population given that the patients presented with metastatic disease and were heavily pretreated prior to the initiation of Phase I therapy. Second, a significant number of patients (39 of 100 referred patients, 39%) were not enrolled on a phase I trial for the most part due to poor overall health and performance status. Finally, mutational analysis was performed in a small subset of patients due to the lack of tissue availability for testing.

Overall, the poor outcomes of advanced HCC patients emphasize the need for new approaches. Therapeutic strategies on clinical trials with inhibitor of angiogenesis and multiple kinases merit further exploration in phase II trials.

## PATIENTS AND METHODS

Of 3614 patients evaluated in the Clinical Center for Targeted Therapy (Phase I Clinical Trials Program) at the M.D. Anderson Cancer Center from November 2004 onwards, we identified 100 consecutive patients with HCC. After review of baseline clinical, laboratory, radiologic and pathologic data during the initial consultation, patients were enrolled on a phase I trial based on scientific rationale and protocol availability. After initiation of an investigational therapy, patients were evaluated at 2- to 4- week intervals, based on the specific protocol, with a history, physical examination, comprehensive series of laboratory tests, and assessments of toxicity and compliance. Restaging scans were done every 6-8 weeks, depending on the protocol.

Specific eligibility criteria varied by protocol; however, common criteria for participation in most phase I clinical trials included presence of metastatic or unresectable disease, measurable disease per Response Evaluation Criteria in Solid Tumors (RECIST), and Eastern Cooperative Oncology Group (ECOG) performance status (PS) of 0-1 (although a PS of 2 is acceptable for certain investigator-initiated studies). This study and all clinical trials were approved by the Institutional Review Board.

### Endpoints and statistical methods

The statistical analysis was performed by our biostatistician (K.R.H.). Descriptive statistics summarized the patients’ characteristics. Cox proportional hazards regression analysis was used to examine the association between progression free survival (PFS) and overall survival (OS) since beginning phase I therapy and the following variables measured at the time of initial Phase I consultation: age at diagnosis, gender, ECOG PS, etiology of liver disease (alcohol abuse, hepatitis B and C, fatty liver disease, etc.), extent of liver disease (cirrhosis, portal hypertension, portal vein thrombosis), number of metastatic sites, number of prior therapies, hemoglobin, lactate dehydrogenase (LDH), platelet count, history of thromboembolism, total bilirubin, aspartate aminotransferase (AST), alanine aminotransferase (ALT), alkaline phosphatase (ALK), serum sodium, serum creatinine, tumor markers (specifically alpha-fetoprotein), and serum albumin.

Best response was assessed using RECIST every 2 cycles (6-8 weeks) of therapy as per the protocol.[12] Partial response (PR) was defined as a >30% decrease in the sum of the longest diameter of target lesions, excluding complete disappearance of disease (complete response, CR) and progressive disease (PD) was a > 20% increase. Stable disease (SD) was defined as changes that did not meet the criteria for a PR or PD. Waterfall plot analysis according to RECIST is used to illustrate response.

Overall survival is defined as the date of enrollment on a Phase I trial until death from any cause or date of last follow-up. PFS is defined as the time from first day of treatment on a phase I trial to date off study of last phase I trial due to disease progression or death, not toxicity. Patients still alive at time of survival analysis and free of progression at time of PFS analysis were censored at time of last follow-up. Toxicities were assessed using the National Cancer Institute Common Terminology Criteria for Adverse Events, version per the protocol. A *P*-value <.05 was considered statistically significant. Statistical analysis was performed using S-PLUS® 8.0 for Windows (Insightful Corp.).
